# Enhanced Wave Absorption and Mechanical Properties of Cobalt Sulfide/PVDF Composite Materials

**DOI:** 10.1038/s41598-019-47037-3

**Published:** 2019-07-19

**Authors:** Qiong Wu, Jian Wu, Guang-Sheng Wang, Hua-Zhao Zhang, Han-Jun Gao, Wei-Jia Shui

**Affiliations:** 1 State Key Laboratory of Virtual Reality Technology and Systems, School of Mechanical Engineering and Automation, Beihang University, Beijing, 100191 P.R. China; 2 Key Laboratory of Bio-Inspired Smart Interfacial Science and Technology of Ministry of Education, School of Chemistry and Environment, Beihang University, Beijing, 100191 P.R. China

**Keywords:** Mechanical engineering, Polymers, Composites, Mechanical properties, Design, synthesis and processing

## Abstract

A cobalt sulfide/PVDF(Polyvinylidene Fluoride) composite was prepared by a simple blending method, and the microstructure of the composite was investigated through X-ray diffraction, scanning electron microscopy, and field emission scanning electron microscopy. Increased absorption properties at frequencies ranging from 2 GHz to 18 GHz were studied, and the mechanical properties were investigated via tensile tests and finite element method simulations. The results indicated that the cobalt sulfide/PVDF composites exhibited strong microwave absorption intensities (−43 dB at 6.6 GHz) with a low filler loading (5.0 wt%). The elastic modulus increased with the cobalt sulfide mass fraction. Cobalt sulfide could improve the mechanical properties of the composite, especially in the lengthwise direction.

## Introduction

As new functional materials, microwave absorbing materials have been broadly applied in the fields of industry, commerce, and military^1,2^. In recent years, porous semiconductor nanomaterials, as the reinforced phase of composite, have widely intrigued researchers due to their good microwave absorption properties, light weight, thin thickness, and good compatibility^3–6^. Good strength is required in practical applications; therefore, the mechanical properties must also be investigated^7,8^.

Many microwave absorbing materials have been reported in the last decade. At present, an increasing number of researchers is focusing on the electromagnetic properties of such materials at frequencies of 2 GHz to 18 GHz^9–13^. Liu *et al*.^14^ prepared a SiC fiber/paraffin wax composite with microwave absorption properties. Its minimum reflection loss (*RL*) was −28.47 dB at 12 GHz. Meng *et al*.^15^ found that SiC microtubes have a minimum RL of −23.9 dB at 17.5 GHz. Meanwhile, a polypyrrole-reduced graphene oxide–Co_3_O_4_ nanocomposite with maximum RL of −33.5 dB at 15.8 GHz was reported by Liu *et al*.^16^. According to Xu *et al*.^17^, smart composite absorbers comprising silicone rubber, multi-walled carbon nanotubes (MWCNTs), and flaky carbonyl iron particles can absorb microwave ranging from approximately 4 GHz to 11 GHz. Many researchers have also investigated PVDF/GIC composites^18^, RGO/CuS/PVDF composites^19^, PVDF/RGO composites^20^, ZnO/PVDF composites^21^, and CuS/PVDF composites^22–24^ in terms of their absorption properties and obtained excellent results. One such finding is that a synergistic effect exists between PVDF and nanofillers^25,26^, and it can improve the wave absorption properties of nanocomposites.

By contrast, CoS nanocomposites and their wave absorption properties have not been researched. A previous study by the present authors revealed that polymer composites filled with inorganic semiconductors exhibit excellent microwave absorption. In this work, we used PVDF as polymeric matrix material for the composite due to its physical and dielectric properties, which are especially suitable for such composite.

Meanwhile, the mechanical performance of certain nano-porous materials has recently been studied by several researchers. Zhao *et al*.^27^ analyzed the effects of carbon fiber (CF) with various content (2%, 4%, 6%, 8%, and 10 wt%) on the mechanical properties of GF/PDMS composites. The experiment data suggested that the addition of CF significantly improved the mechanical properties of the GF/PDMS composites. The composite with 10 wt% CF (F/GF/PDMS composite) evidently increased in tensile strength (by 52%) and Young’s modulus (by 71%). Rajaei *et al*.^28^ studied how ammonium olyphosphate and talc affect the mechanical property of an epoxy/glass fabric composite. With the addition of flame retardants to the epoxy resin, the composite’s tensile and flexural moduli increased. Shin *et al*.^29^ investigated the mechanical properties of aluminium alloy 2024 (Al2024) matrix composites that were individually reinforced with either an MWCNT or few-layered graphene at 250 °C to 430 °C. Results showed that both resultant composites formed at 350 °C possessed high yield stress of approximately 110 MPa. Otto *et al*.^30^ analyzed the mechanical properties of several low-cost hybrid composites consisting of polyurethane and renewable natural fibers. A simplex-centroid mixture design model was used to evaluate the effects of the added fibres on composite properties, such as resilience, elastic modulus, and deformation, under permanent compression. The obtained hybrid composites demonstrated excellent performances of up to 32% resilience, 0.1 GPa elastic modulus, and 7.32% permanent deformation. Tarfaoui *et al*.^31^ investigated the relationship between the addition of carbon nanotube (CNT) additives and the elasticity of textile-based composites. The experimental results indicated that the mechanical properties of the composites increased with the CNT additive content and reached its peak value at 2%.

In this research, cobalt sulfide/PVDF composites were prepared using a simple blending method and then studied with respect to their microstructure through scanning electron microscopy (SEM), X-ray diffraction (XRD), and X-ray photoelectron spectroscopy. The improved wave-absorption ability of cobalt sulfide/PVDF composite was studied, and the mechanical properties were explored via tensile tests and finite element method (FEM) simulations.

## Experiments

### Preparation of CoS nano-porous materials

Cobalt chloride hexahydrate (CoCl_2_ · 6H_2_O, analytical reagent) was obtained from Guangdong Guanghua Sci-Tech Co., Ltd. (China), and ethylenediamine (EDA, AR) was supplied by Xilong Chemical Co., Ltd. (China). N, N-dimethyl formamide (DMF, AR), sulfur powders (S, AR), glycol (HOCH_3_CH_2_OH, AR), absolute ethanol (CH_3_CH_2_OH, AR), and deionized water (H_2_O, AR) were acquired from Beijing Chemical Reagents Co. (China).

1-D cobalt sulphide nanomaterials were prepared first. A total of 1.2 mmol of cobalt chloride hexahydrate and 1.2 mmol of sulfur powders were dissolved in 135 ml ethylene glycol by the ultrasonic dissolving method. After 1 h magnetic stirring, the cobalt chloride hexahydrate was completely dissolved, and the sulfur powders were uniformly dispersed in the flask. Then, 15 ml ethylenediamine was added, and magnetic stirring was performed to uniformly disperse the ethylenediamine while the flask was maintained at 120 °C for 6 h in an oil bath. Eventually, orange CoS powders were obtained after centrifugal separation, absolute ethanol cleaning, and heat treatment with nitrogen protection in sequence.

**Figure 1:**
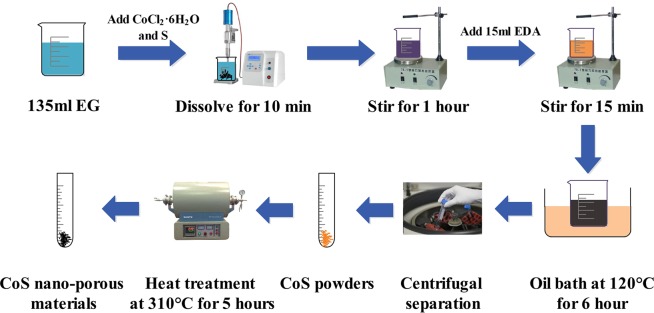
Synthesized process of CoS nano-porous materials.

Subsequently, the obtained orange solid powders were spread uniformly into a crucible in a tube furnace. The temperature was raised from 50 °C to 310 °C in 240 min with the nitrogen protection. The temperature was maintained at 310 °C for 5 h and then reduced to room temperature. Finally, black powders were obtained (Fig. 1).

### Preparation of CoS/PVDF composites

**Figure 2:**
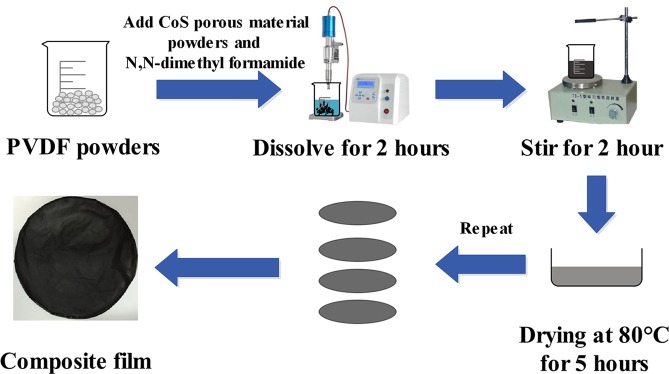
Synthesized process of CoS/PVDF composites.

The cobalt sulfide/PVDF composites were established by mixing 1-D nano-porous cobalt sulfide powders and polymer PVDF through blending and hot moulding (Fig. 2). The nano-porous cobalt sulfide powders and PVDF were placed in a beaker. Mixtures with 5%, 10%, 20%, and 30% cobalt sulfide powders were prepared. Then, N, N-dimethyl formamide was added in the breaker. The cobalt sulfide powders were uniformly dispersed and the PVDF was completely dissolved after 2 h ultrasonic and magnetic stirring (Supplementary Fig. S1). Afterwards, the mixture was placed in an oven at 80 °C for 4 h for drying. Then, the cobalt sulfide/PVDF composite film was obtained (Supplementary Fig. S1). Subsequently, the dried mixture was collapsed and then compressed into wafers at 220 °C and 6 MPa pressure for 10 min. Then, it was cooled to ambient temperature under the same pressure.

### Characterization

XRD analysis was conducted to identify the crystal structures of the cobalt sulfide powders using a Shimadzu 6000 X-ray diffractometer with Cu Kα radiation under an accelerating voltage of 15 kV. Refer to the norm of experimental characterization (Joint Committee on Powder Diffraction Standards Card No. 65-3418), the grain morphology and size were analyzed through SEM and field emission SEM (FESEM) using a JEOL JSM-7500F microscope. Relative permittivity *e* was measured by an Anritsu 37269D Vector Network Analyzer in the range of 2 GHz to 18 GHz using the coaxial probe method. Then, reflection loss at different electromagnetic wave frequencies could be calculated.

### The numerical model

**Figure 3:**
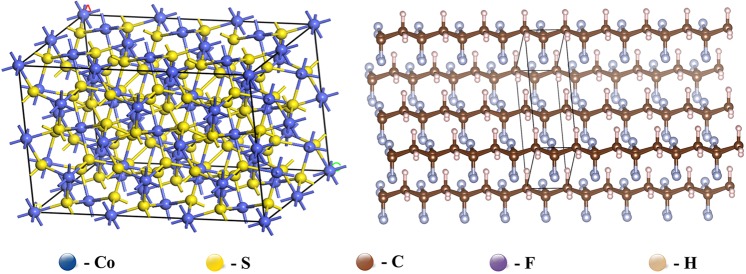
Molecular structure of CoS and PVDF.

The molecular structure of cobalt sulfide and PVDF were established to obtain the mechanical properties of the reinforcement and matrix (Fig. 3). The parameters of the lattice for CoS are a = 3.37 nm, b = 2 nm and c = 3 nm. And the parameters of the lattice for PVDF are a = 0.86 nm, b = 0.49 nm and c = 0.25 nm. The simulation results of mechanical properties (Elastic modulus and Poisson ratio) are shown in Table 1.

**Table 1:** Mechanical properties in FEM simulations.

Parameters	PVDF	CoS
Elastic modulus (MPa)	840	300000
Poisson ratio	0.2	0.3
Density (kg/m^3^)	1800	5450

**Figure 4:**
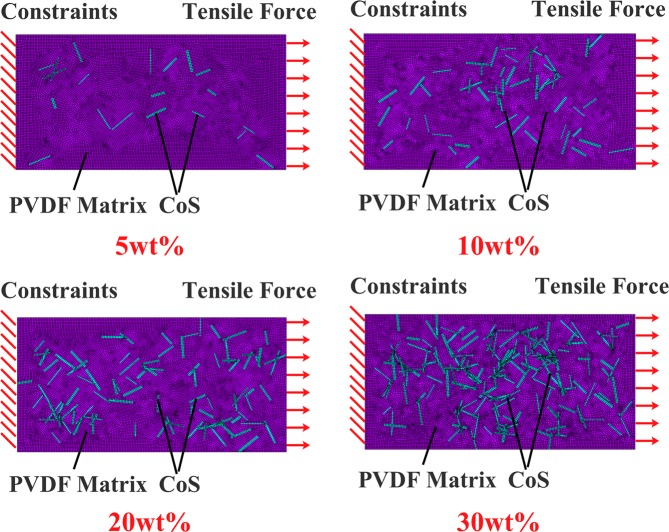
FE models of CoS/PVDF composite.

FEM simulations were performed to further study the composite with respect to its mechanical properties. The FE models of the 5 wt%, 10 wt%, 20 wt%, and 30 wt% cobalt sulfide/PVDF composites are shown in Fig. 4.

The numerical model mainly includes the following steps: (1) Create a geometric model of the composite. A 100 × 50 × 5 μm rectangular shell was built to stand for the PVDF matrix in the software ANSYS FEM. (2) Define a random distribution of cobalt in the matrix. Several small internal rectangles with different sizes and positions represented the nano-porous cobalt sulfide. The nano-porous cobalt sulfide ranges in length from 4 μm to 10 μm and in width from 0.4 μm to 1 μm. The random distribution of cobalt sulfide is based on the modeling algorithm (Supplementary Fig. S2). (3) Define material properties. The mechanical properties identified in the FEM simulation are shown in Table 1. (4) The hexahedral element is used for mesh division, and the size of the element is 0.6 μm. The number of the finite element in the numerical model is 90765 and the type of element is SOLID185. (5) Define the load and boundary conditions. One short edge of the PVDF matrix was constrained, and the tensile stress (40 MPa) was applied to the other short edge. Due to the limitation of finite element method, the contact between cobalt sulfide and PVDF is set as a tight binding. Thus, the relationship between strain and stress was calculated through static analysis, and the elastic modulus of the composite could be determined.

### Mechanical tests

To investigate mechanical properties of the cobalt sulfide/PVDF composites with 5 wt%, 10 wt%, 20 wt%, and 30 wt% (weight fraction) cobalt sulphide, mechanical tests were carried out according to ASTM D882-12 standard. A 1000 N universal testing machine (Shimadzu AGS-X) was used at a loading speed of 50 mm/min. The thickness of the composite film, which was cut into several 9 mm × 3 mm rectangular pieces, was 55 μm. Two pieces of paper were pasted to both ends of each rectangular specimen for clamping of the tensile test (Supplementary Fig. S3). Six groups of composite materials with the same mass fraction were prepared for six tensile tests. The experimental details of mechanical tests are shown in Table 2. In this manner, the stress–strain relationship of the composite could be obtained.

**Table 2:** Mechanical test experimental parameters.

Equipment	Temperature	Relative humidity	Loading speed	Position detection resolution
Shimadzu AGS-X	25 °C	65%RH	50 mm/min	0.033 μm

## Results and Discussion

### Morphology and structural analysis of samples

**Figure 5:**
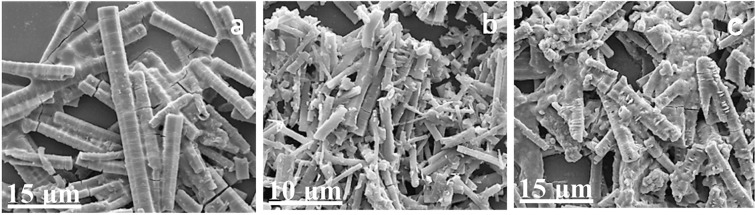
SEM images of CoS product obtained using different molar ratios of CoCl_2_ · 6H_2_O: S (**a**) CoCl_2_ · 6H_2_O/S = 1:1, (**b**) CoCl_2_ · 6H_2_O/S = 1:2, and (**c**) CoCl_2_ · 6H_2_O/S = 2:1.

Figure 5 shows that the CoS product can be described as a mixture of nanorods and nanotubes with length of 15 μm to 50 μm and a pentagonal or hexagonal section. In this reaction, Rs (the molar ratio of CoCl_2_ · 6H_2_O/S) is a key factor that affects the prepared product structure. When Rs is 1:1, nanorods with uniform morphologies are obtained (Fig. 5a). When Rs decreases to 1:2 (Fig. 5b), a large number of nanorods is clumped together and shows uneven diameters. The lengths of the nanorods decrease from 15 μm to 300 nm with an increase in Rs. When Rs increases to 2:1 (Fig. 5c), the nanorods are thick, and brittle fracture appears.

The amino groups at both ends of the ethylenediamine can chelate metal ions due to the special structure. Therefore, the growth mechanism of the 1-D cobalt sulfide nanomaterials can be explained as follows. The amino groups at both ends of the ethylenediamine chelate metal ions, and the chelation makes cobalt sulfide grow along both ends of the ethylenediamine.

**Figure 6:**
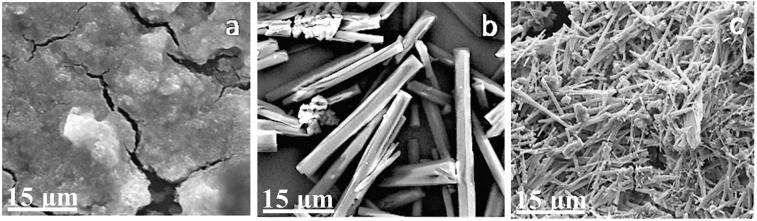
CoS product under different oil bath temperatures: (**a**) 140 °C for 6 h, (**b**) 120 °C for 6 h, and (**c**) room temperature for 12 h.

**Figure 7:**
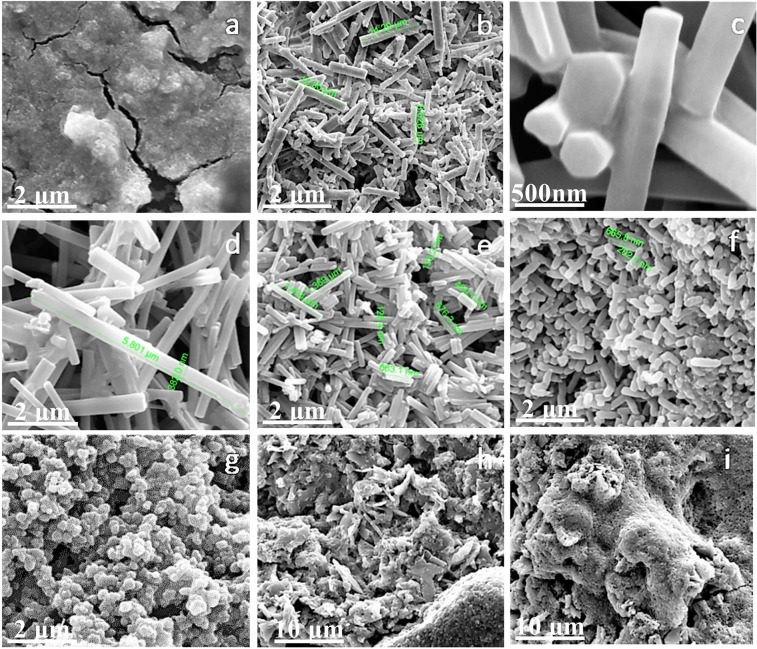
CoS product under different EDA/EG: (**a**) EDA/EG = 1/149; (**b**,**c**) EDA/EG = 1/9; (**d**) EDA/EG = 1/5; (**e**) EDA/EG = 1/2; (**f**) EDA/EG = 1/1; (**g**) EDA/EG = 2/1; (**h**) EDA/EG = 5/1; (**i**) pure EDA.

Figure 6b shows that the optimal temperature for the oil bath is 120 °C. Figure 6a shows that agglomerations of the product appear under a high temperature (140 °C). Figure 6c presents that the growth rate of the product evidently decreases and the product becomes increasingly slender and brittle under a low temperature (room temperature). Figure 7 shows that the length of the product decreases with an increase in the ratio of ethylene diamine and ethylene glycol (EDA/EG). The length ranges from 15 μm to 0.3 μm, and the section of the CoS product is pentagonal or hexagonal (Fig. 7c). The product is spherical when EDA/EG is 2:1 (Fig. 7g). The product has no fixed morphology and is aggregated into an amorphous block in pure ethylene diamine (Fig. 7i). The reaction does not occur in pure ethylene glycol.

**Figure 8:**
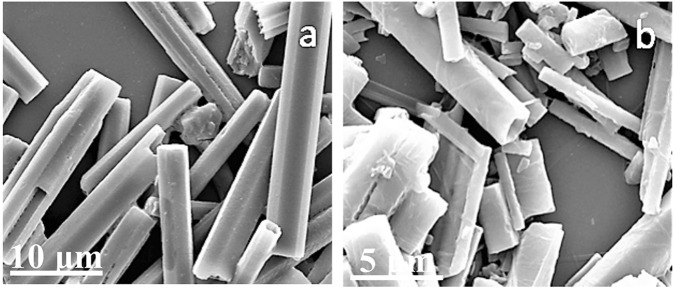
SEM images of typical CoS nanomaterial.

**Figure 9:**
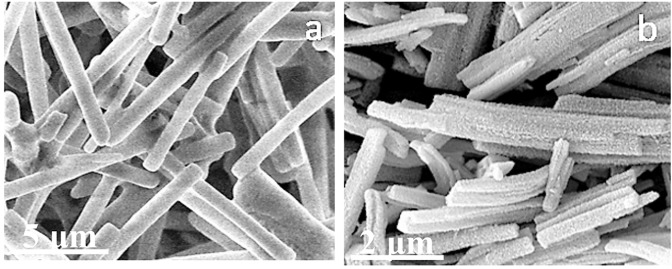
SEM images of CoS (**a**) before heat treatment and (**b**) after heat treatment.

The SEM images in Fig. 8 present the typical structure of the product. SEM images of CoS before and after 310 °C heat treatment are presented in Fig. 9a,b, respectively. Small holes that measure several nanometers in diameter, which improve the wave absorption properties, appear on the nanorods after 310 °C heat treatment for 5 h. An energy-dispersive spectroscopy chart of the CoS porous material is shown in Supplementary Fig. S4.

**Figure 10:**
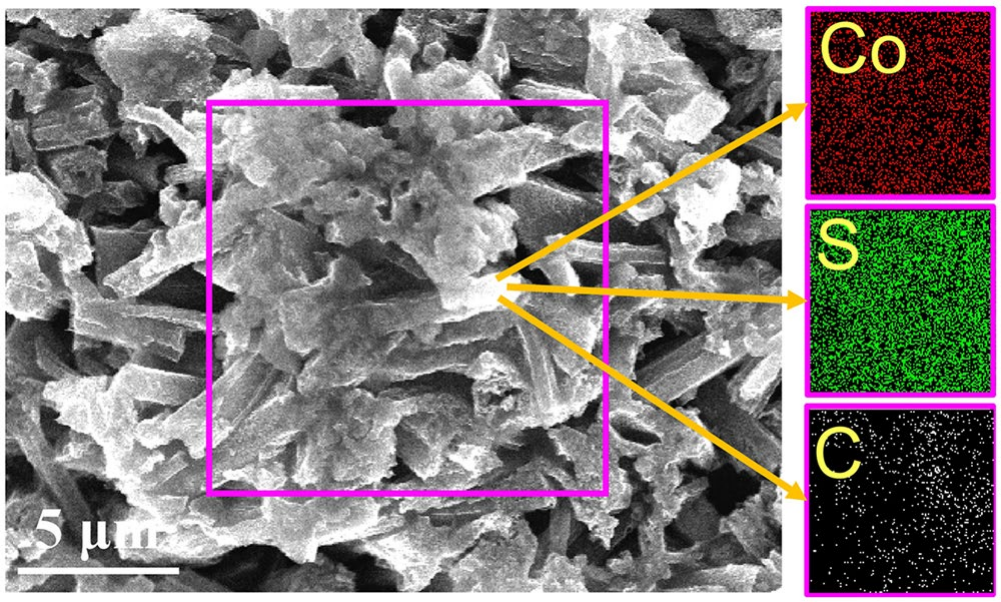
FESEM images of cobalt sulfide/PVDF membrane and elemental maps of Co, S, and C in composites.

**Figure 11:**
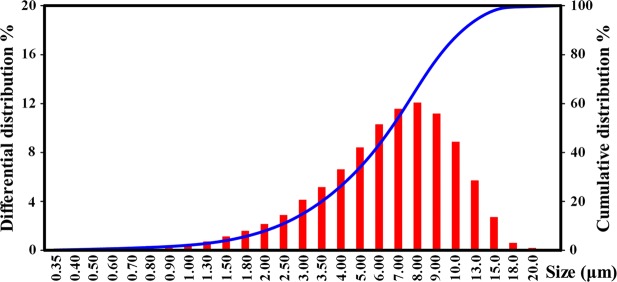
Distribution curve of cobalt sulfide size.

As illustrated in Fig. 10, the FESEM image of the composite indicates that the cobalt sulfide particles are well dispersed in the polymer, thereby generating a compact structure. The distribution curve of cobalt sulfide is shown in Fig. 11. Moreover, the FESEM image shows that the cobalt sulfide particles dispersed in the composite maintain their morphology. According to the FESEM characterization data and the cobalt sulfide/PVDF elemental maps provided in the same figure, the elements of Co and S that are on the surface of the nanocomposites demonstrate that the cobalt sulfide is well dispersed in the PVDF. The excellent distribution of the Co and S atoms in the polymer can also be confirmed through area scan analysis in the rectangular region. The good dispersion of the cobalt sulfide in the PVDF may benefit the mechanical and wave absorption properties of the polymer.

### Absorption properties

The wave absorption properties were measured by reflection loss (*RL*), which can be expressed as follows^32^:

1 $$RL=20\,\mathrm{log}|\frac{{Z}_{in}-1}{{Z}_{in}+1}|,$$

2 $${Z}_{in}=\sqrt{\frac{{\mu }_{r}}{{\varepsilon }_{r}}}\,\tanh [\,j\,(\frac{2f\pi d}{c})\sqrt{{\mu }_{r}{\varepsilon }_{r}}],$$

where *Z*_*in*_ stands for input impedance, *μ*_*r*_ stands for relative complex permeability (The *μ*_*r*_ of cobalt sulfide is approximately equal to 1), *ε*_*r*_ stands for complex relative permittivity, *f* stands for the microwave frequency, *d* stands for absorbent’s thickness, *h* stands for the Planck constant, and *c* stands for the propagation velocity of an electromagnetic wave in vacuum.

In this work, relative permittivity was measured by the Anritsu 37269D Vector Network Analyzer in the range of 2 GHz to 18 GHz using the coaxial probe method.

**Figure 12:**
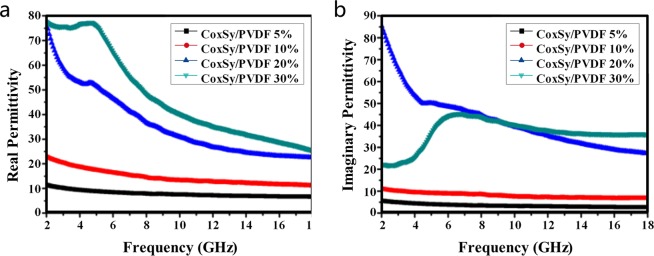
Measured frequency dependence of (**a**) real parts and (**b**) imaginary parts of dielectric permittivity.

The frequency dependence of the relative permittivity was studied, and the results are provided in Fig. 12. The real permittivity *ε*′ of the cobalt sulfide/PVDF composites is higher than that of pure PVDF (approximately 3.0 at 2 GHz) and generally proportional to the load content (Fig. 12a), except a small decrease in the range of 2 GHz to 18 GHz for all involved sulfide/PVDF composites with any load content, which is the same as the imaginary permittivity *ε*″ (Fig. 12b). COMSOL Multiphysics has obvious advantages in the multi-physics coupling analysis, and the frequency dependence of the relative permittivity can be simulated numerically based on COMSOL Multiphysics. According to the data presented in Fig. 12, the dielectric loss tangent tan(*σ*) *ε*″/*ε*′ of the composites with different filler loadings was calculated; on this basis, the theoretical *RL*s of the PVDF and cobalt sulfide/PVDF composites with filler loadings of 5 wt%, 10 wt%, 20 wt%, and 30 wt% at 2.5 mm thickness could be obtained through Eqs (1) and (2).

**Figure 13:**
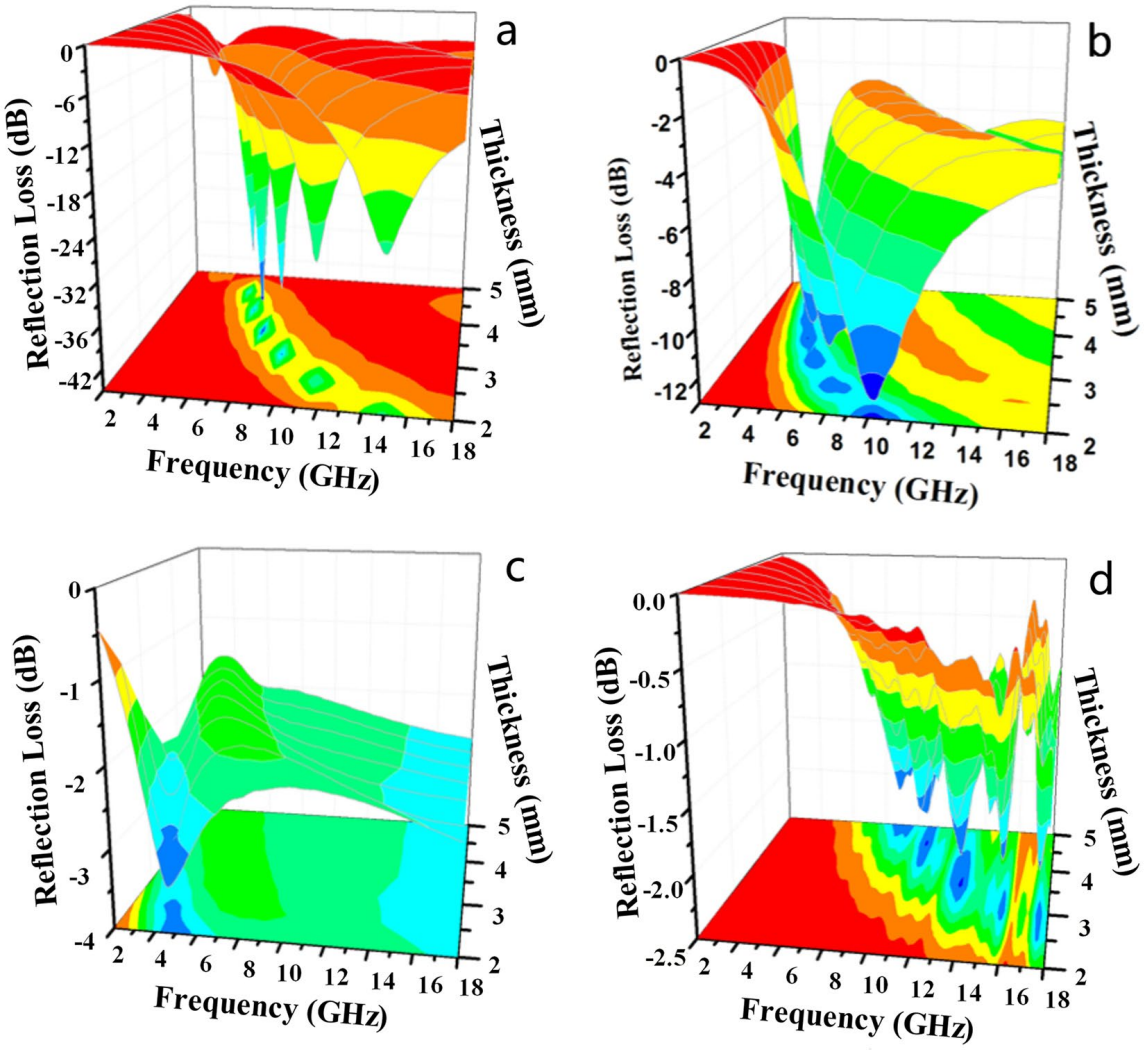
3-D presentations of RL of cobalt sulfide/PVDF composites with loadings of (**a**) 5 wt%, (**b**) 10 wt%, (**c**) 20 wt%, and (**d**) 30 wt.

The 3-D and 2-D presentations of *RL*, which (Fig. 13) indicate the calculated theoretical *RL*s of the various cobalt sulfide/PVDF composites in the range of 2 GHz to 18 GHz are provided in Fig. 13, covering the composites with various thicknesses (2 mm to 5 mm) and different loadings of 5 wt%, 10 wt%, 20 wt%, and 30 wt%. The results suggest that microwave absorption properties and the minimum *RL*s corresponding to the peak absorptions, which gradually appear at various frequencies in the range of 2 GHz to 18 GHz by regulating the absorbers’ thickness, may not be practically achievable. With respect to the loading content, the absorption of the cobalt sulfide/PVDF composites peaks at 5 wt%, and then it gradually decreases with an increase in loading up to 20 wt%. For the composites with 5 wt% cobalt sulfide, the sharpest peak, which is up to −43 dB at 6.60 GHz, occurs when the thickness of the absorbers is 4.3 mm; stronger peaks could be acquired by adjusting the absorbers’ thickness.

The Debye dipolar relaxation plays a significant role in absorbing microwave for dielectric-loss materials. Its relative complex permittivity is expressed as Eq. (3)^33^

3 $${\varepsilon }_{r}={\varepsilon }_{\infty }+\frac{{\varepsilon }_{s}-{\varepsilon }_{\infty }}{1+j2\pi f\tau }=\varepsilon ^{\prime} -j\varepsilon ^{\prime\prime} ,$$

where *f* stands for the frequency, *ε*_*s*_ stands for static permittivity, *ε*_*∞*_ stands for relative dielectric permittivity at high-frequency limit, and the *τ* stands for polarization relaxation time. Therefore, *ε*′ and *ε*″ can be described by

4 $$\varepsilon ^{\prime} ={\varepsilon }_{\infty }+\frac{{\varepsilon }_{s}-{\varepsilon }_{\infty }}{1+{(2\pi f)}^{2}{\tau }^{2}},$$

5 $$\varepsilon ^{\prime\prime} =\frac{2\pi f\tau ({\varepsilon }_{s}-{\varepsilon }_{\infty })}{1+{(2\pi f)}^{2}{\tau }^{2}}.$$

The relationship between *ε*′ and *ε*″ is obtained as follows by reducing Eqs (4) and (5):

6 $${(\varepsilon ^{\prime} -\frac{{\varepsilon }_{s}+{\varepsilon }_{\infty }}{2})}^{2}+{(\varepsilon ^{\prime\prime} )}^{2}={(\frac{{\varepsilon }_{s}-{\varepsilon }_{\infty }}{2})}^{2}.$$

Therefore, the *ε*′ − *ε*″ curve will be a single semicircle. The semicircle is denoted as the Cole–Cole semicircle in general, and each semicircle and one Debye relaxation process share a one-to-one correspondence.

The *ε*′ − *ε*″ plots of the cobalt sulfide/PVDF with various content of cobalt sulfide, which are in semicircles, are presented in Supplementary Fig. S5. Interfacial polarization or the Maxwell–Wagner effect is observed due to the functional groups on the surface of the cobalt sulfide and interfaces in the cobalt sulfide/PVDF composites.

An increase in the composite’s conductivity can lead to significant dielectric loss according to free electron theory. Hence, the increased filler content of cobalt sulfide should increase the dielectric loss of the cobalt sulfide/PVDF composite because the superior conductivity of the cobalt sulfide can increase the conductivity of the composites.

### Mechanical properties

**Figure 14:**
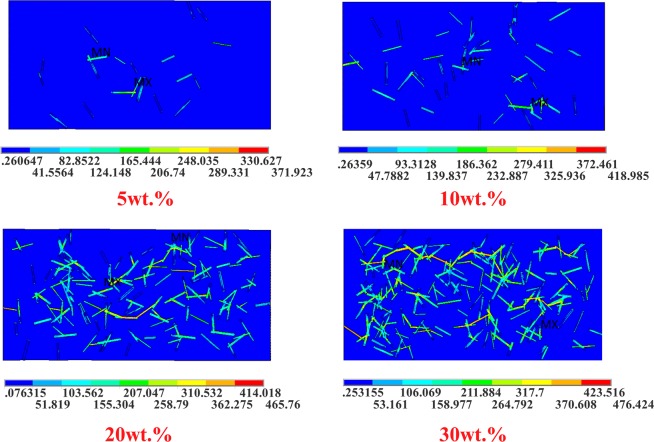
Equivalent stress results of FE model.

**Figure 15:**
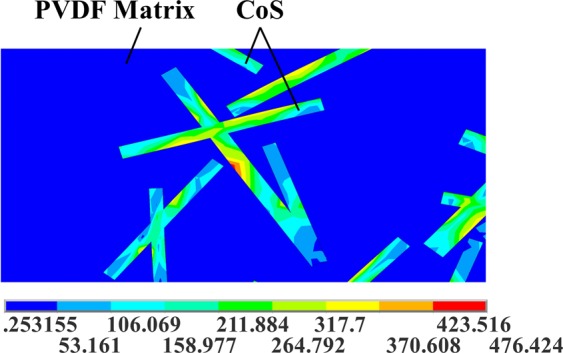
Equivalent stress results of FE model (Partial enlargement).

The equivalent stress (von Mises stress) results of the FE model are represented in Fig. 14, and the partial enlargement of the equivalent stress of 20 wt% is shown in Fig. 15. The stress in the cobalt sulfide is considerably larger than that in the PVDF matrix. The FE model results of cobalt sulfide/PVDF composites show that the stress of the matrix is small (less than 50 MPa), but the stress of the reinforcement is large (more than 150 MPa). Moreover, with the increase of the mass fraction of cobalt sulfide, the maximum stress of cobalt sulfide/PVDF composite increases. The former increases with a decrease in the included angle between the tensile direction and the cobalt sulfide lengthwise direction. Therefore, cobalt sulfide can improve the mechanical properties of the composite, especially in the lengthwise direction.

**Figure 16:**
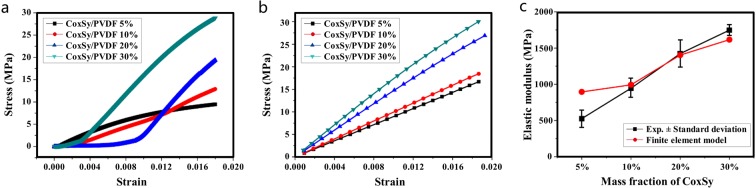
Stress–strain curve by tensile tests and FEM simulation: (**a**) tensile test, (**b**) FEM simulation, (**c**) comparison between measured and calculated elastic modulus.

The stress–strain curves of the elastic stage obtained by tensile tests and FEM simulations are shown in Fig. 16a,b, and the elastic modulus results are presented in Fig. 16c. The stress contours of FEM simulation are shown in Fig. 16.

The elastic modulus of cobalt sulfide and PVDF are 300 GPa and 840 MPa, respectively. For cobalt sulfide/PVDF Composite Materials, under the condition of the same strain, the elastic modulus of cobalt sulfide is bigger, so the equivalent stress of cobalt sulfide is larger. With the addition of cobalt sulfide, the elastic modulus of cobalt sulfide/PVDF material increased significantly. The measured elastic modulus of 5 wt%, 10 wt%, 20 wt%, and 30 wt% is 524.73, 944.98, 1422.2, and 1746.6 MPa, respectively. The elastic modulus calculated by the FE model is 895.62, 991.71, 1484.29, and 1784.09 MPa, respectively. These results show that the elastic modulus increases with the cobalt sulfide mass fraction. Therefore, cobalt sulfide is conducive to the improvement of the composites’ mechanical properties. The consistency between the FEM results and the experimental results validates the effectiveness of FEM.

## Conclusions

In this research, two-phase composites comprising cobalt sulfide and PVDF were synthesized and studied in terms of their microstructures and characterization, and the wave absorption and mechanical properties were explored. The results suggested that the cobalt sulfide/PVDF composites have strong microwave absorption intensity, reaching −43 dB at 6.6 GHz. This intensity can be tuned by regulating the thickness and the content levels of the cobalt sulfides. Moreover, the elastic modulus is increased with the cobalt sulfide mass fraction. With all the abovementioned improvements and advantages, the cobalt sulfide/PVDF composites are still as flexible as pure PVDF and also can be cut into desired morphologies. Therefore, cobalt sulfide is conducive to the improvement of the mechanical properties of composites. The cobalt sulfide/PVDF composites can be applied in commercial, military, and scientific electronic devices due to their outstanding microwave absorption performance and potential for industrial production.

## Supplementary information

Supporting Information
