# Supplementary material for: Enhanced Wave Absorption and Mechanical Properties of Cobalt Sulfide/PVDF Composite Materials
Source: Sci Rep. 2019 Jul 19;9:10488. doi: 10.1038/s41598-019-47037-3 (PMC6642107; doi:10.1038/s41598-019-47037-3)
Supplement: Supplementary file 1 — Supporting Information [file 41598_2019_47037_MOESM1_ESM.pdf]

Supporting Information

Enhanced Wave Absorbing and Mechanical Properties of Cobalt Sulfide/PVDF Composite Materials

Qiong Wu[a], Jian Wu[a], Guang-Sheng Wang[b,*], Hua-Zhao Zhang[a], Han-Jun Gao[a] and Wei-Jia Shui[a]

[a] *State Key Laboratory of Virtual Reality Technology and Systems, School of Mechanical Engineering and Automation, Beihang University, Beijing, 100191, PR China;*

[b] *Key Laboratory of Bio-Inspired Smart Interfacial Science and Technology of Ministry of Education, School of Chemistry and Environment, Beihang University, Beijing 100191, PR China.*

Supplementary Figures

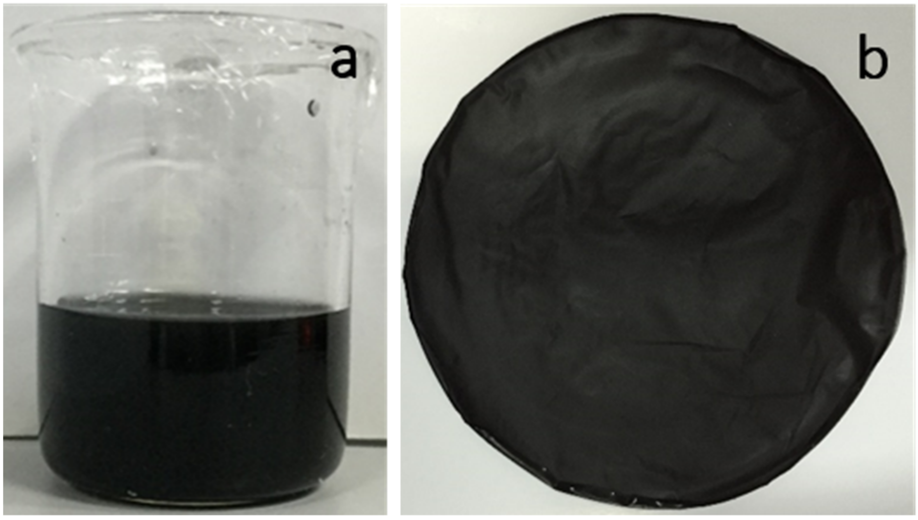

Supplementary Fig. S1 Preparation of Cobalt Sulfide /PVDF Composite. (a) mixed solution of cobalt sulfide and PVDF in DMF (b) cobalt sulfide/PVDF composite film.

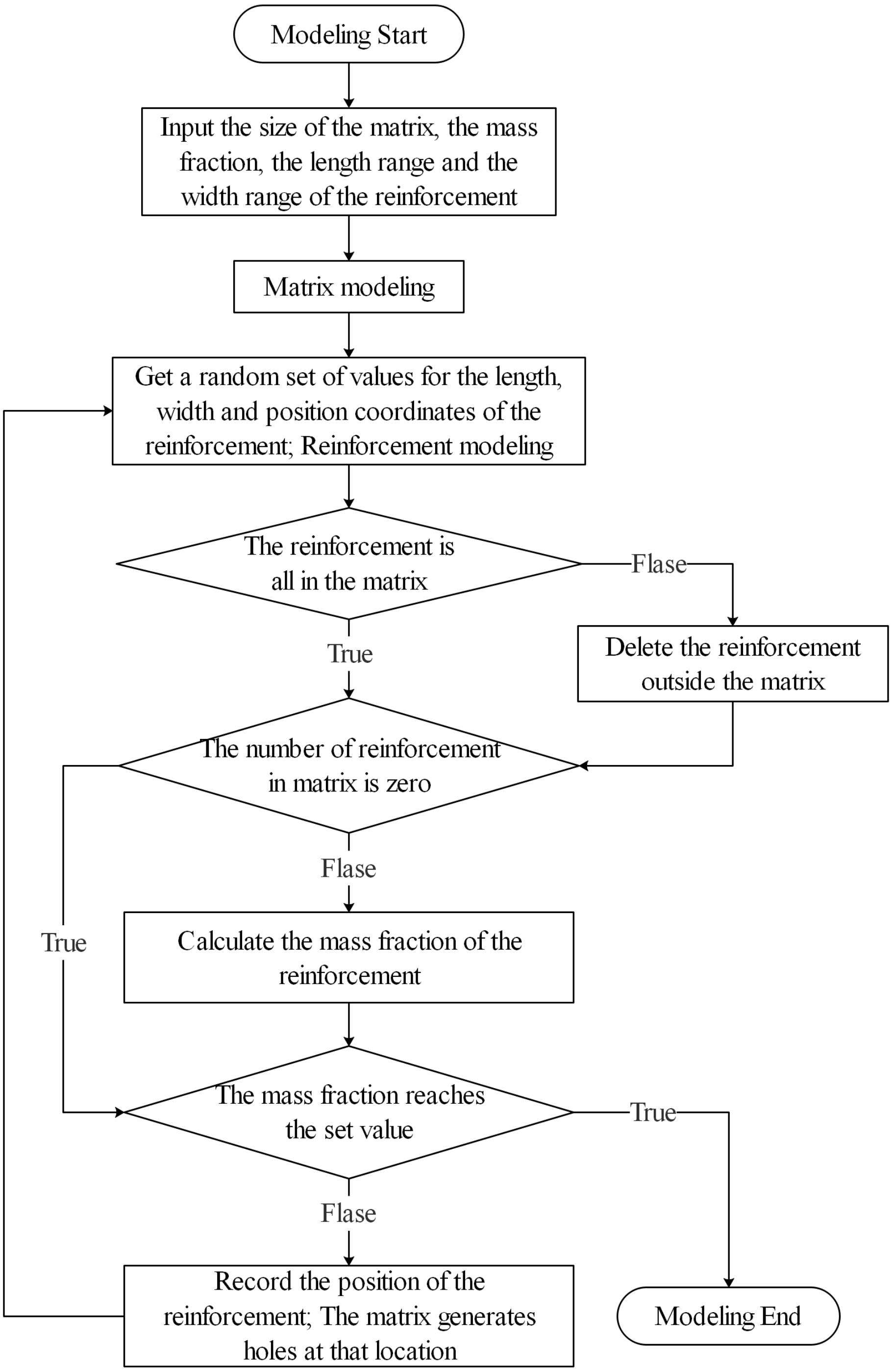

Supplementary Fig. S2 Cobalt sulfide random distribution algorithm.

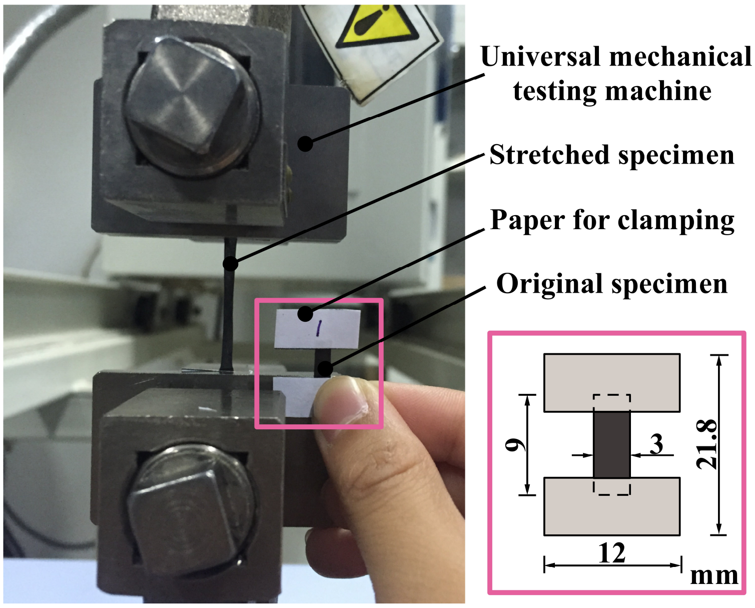

Supplementary Fig. S3 Tensile tests.

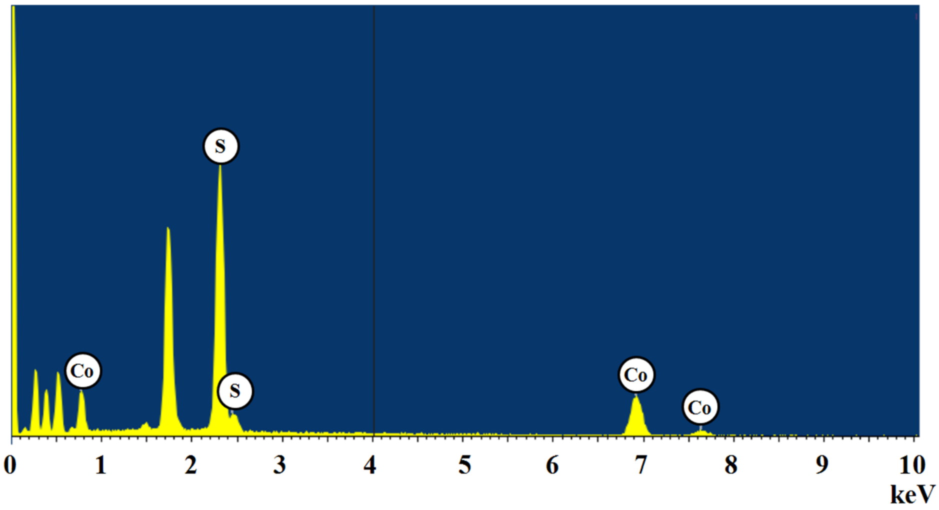

Supplementary Fig. S4 Energy Dispersive Spectroscopy (EDS) chart of CoS porous material.

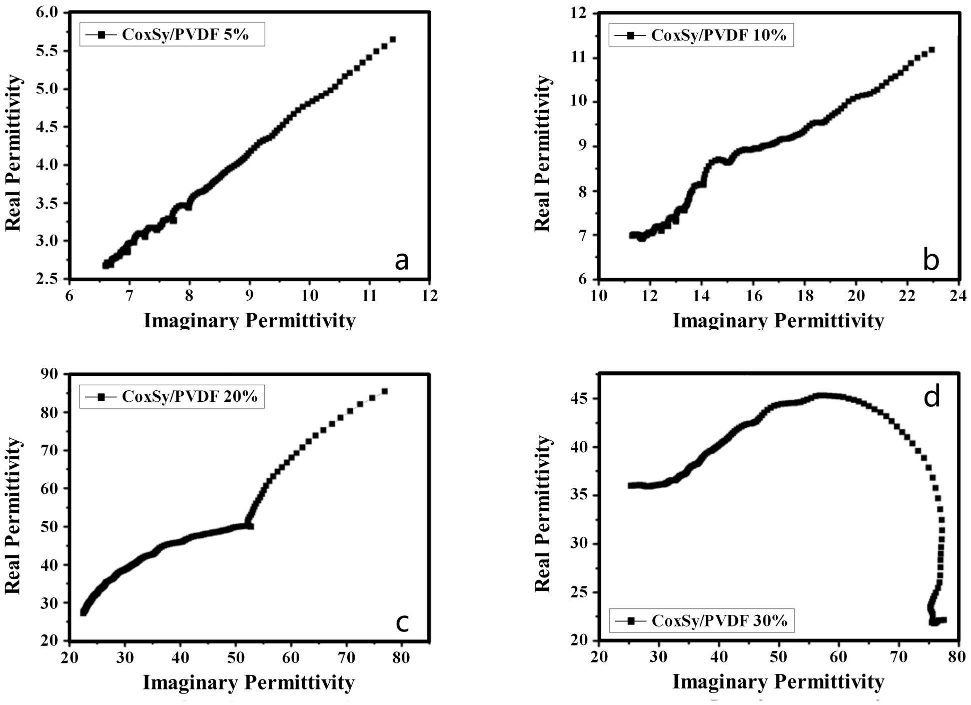

Supplementary Fig. S5 ε'-ε" curves of (a) filler loading of 5 wt%; (b) filler loading of 10 wt%; (c) filler loading of 20 wt% and (d)filler loading of 30wt%.
